# Exploring *Phaeodactylum tricornutum* for Nutraceuticals: Cultivation Techniques and Neurotoxin Risk Assessment

**DOI:** 10.3390/md23020058

**Published:** 2025-01-26

**Authors:** Tobias Ebbing, Lena Kopp, Konstantin Frick, Tabea Simon, Berit Würtz, Jens Pfannstiel, Ulrike Schmid-Staiger, Stephan C. Bischoff, Günter E. M. Tovar

**Affiliations:** 1 Fraunhofer Institute for Interfacial Engineering and Biotechnology, 70569 Stuttgart, Germany; konstantin.frick@igb.fraunhofer.de (K.F.); tabea.simon@igb.fraunhofer.de (T.S.); ulrike.schmid-staiger@igb.fraunhofer.de (U.S.-S.); guenter.tovar@igvp.uni-stuttgart.de (G.E.M.T.); 2 Institute of Interfacial Process Engineering and Plasma Technology (IGVP), University of Stuttgart, Pfaffenwaldring 31, 70569 Stuttgart, Germany; 3 Institute of Nutritional Medicine, University of Hohenheim, Fruwirthstr. 12, 70593 Stuttgart, Germany; lena.stiefvatter@uni-hohenheim.de (L.K.); bischoff.stephan@uni-hohenheim.de (S.C.B.); 4 Core Facility Hohenheim, Mass Spectrometry Unit, University of Hohenheim, Ottlie-Zeller-Weg 2, 70599 Stuttgart, Germany; berit.wuertz@uni-hohenheim.de (B.W.); jens.pfannstiel@uni-hohenheim.de (J.P.)

**Keywords:** microalgae, *Phaeodactylum tricornutum*, diatom, nutrition, flat-plate airlift photobioreactor, β-methylamino-L-alanine

## Abstract

This study investigates the potential of the diatom *Phaeodactylum tricornutum* (PT) as a sustainable and nutritionally valuable food source, focusing on its ability to produce bioactive compounds such as eicosapentaenoic acid, fucoxanthin, chrysolaminarin (CRY) and proteins. PT was cultivated in a flat-plate airlift photobioreactor (FPA-PBR) illuminated with LEDs from two sides. The study aimed to monitor and minimize β-methylamino-L-alanine (BMAA) levels to address safety concerns. The data showed that the selected FPA-PBR setup was superior in biomass and EPA productivity, and CRY production was reduced. No BMAA was detected in any biomass sample during cultivation. By adjusting the cultivation conditions, PT biomass with different compositional profiles could be produced, enabling various applications in the food and health industries. Biomass from nutrient-repleted conditions is rich in EPA and Fx, with nutritional and health benefits. Biomass from nutrient-depleted conditions accumulated CRY, which can be used as dietary fiber. These results highlight the potential of PT as a versatile ingredient for human consumption and the effectiveness of FPA-PBRs with artificial lighting in producing high-quality biomass. This study also provides the basis for future research to optimize photobioreactor conditions to increase production efficiency and to tailor the biomass profiles of PT for targeted health-promoting applications.

## 1. Introduction

The impact of population growth and climate change on the food sector is significant and requires a reassessment of future diets and production systems [1]. To meet these challenges sustainably and reduce pressure on global resources, it is essential to develop innovative foods with optimized nutrient profiles, such as insects, fungi, lab-grown meat or algae [2]. Among these, algae stand out due to their diverse properties and long history of use as food. They can be divided into macroalgae and microalgae based on their size and cell structure. Microalgae are single-celled organisms known for their potential as cell factories for valuable products [3]. An interesting microalga is the diatom *Phaeodactylum tricornutum*, which is highly effective in synthesizing proteins; eicosapentaenoic acid (EPA), an omega-3-fatty acid; fucoxanthin (Fx); and chrysolaminarin (CRY), making it a strong candidate for food applications [4]. In detail, PT can be a significant source of protein, offering up to 60% of its dry weight, with a favorable amino acid composition. Studies in mice have demonstrated that up to 48% of the protein in their diet can be replaced with PT biomass [5]. Additionally, PT is a primary producer of the omega-3 fatty acid EPA, providing a sustainable alternative to fish, potentially addressing the growing global demand for EPA [6]. EPA is also well known for its anti-inflammatory properties [7]. Beyond EPA, PT is rich in beneficial carotenoids, such as Fx and β-carotene, which are linked to obesity reduction and vision health [8,9]. The production of these bioactive compounds adjusts PT’s cultivation conditions. Under nutrient-repleted growth conditions, PT produces high amounts of EPA and Fx. With nitrogen depletion conditions in the photobioreactor, PT accumulates CRY as carbon and energy storage [10,11]. CRY is a water-soluble β-1,3-glucan, which has been shown to promote gut health and improve antioxidant status in both animals and humans [12,13,14]. This biomass could be used in food applications, as it has promising health benefits, including cholesterol-lowering properties [15]. The stated studies utilized PT biomass produced in flat-plate airlift photobioreactors (FPA-PBRs) with artificial illumination from one side and stressed the suitability of this setup to yield superior productivities for EPA, FX and CRY. Nevertheless, studies investigating the fundamentals of photobioreactor design and development have stressed the importance of illuminated surface and a reduction in non-illuminated volumes of a photobioreactor to increase the productivity of high-quality biomass [16,17].

Due to these properties, PT is already used in fish farming, replacing up to 6% of fishmeal [18]. For applications in human nutrition, however, it is necessary to mechanically break the thick cell wall of PT to ensure the bioavailability of the nutrients [19]. Due to its unique physicochemical and functional properties, PT also has the potential to be used as a structuring agent in food [20]. While PT is not yet approved as a novel food within the European Union and is regulated under Novel Food Regulation (EU) 2018/456, commercial products featuring PT are already available, such as Algatech’s FucoVital™, marketed for liver health, and various offerings from Mycrophyt. For PT to gain approval as a novel food in the EU, a comprehensive application must be submitted, detailing aspects such as the manufacturing process, intended uses and toxicological data [21]. Although there is increasing evidence that PT is suitable for human consumption, further validation of its safety is required. Currently, PT is not eligible for the qualified presumption of safety (QPS) status, primarily due to its limited history of safe use in the food chain and its potential to produce bioactive compounds such as β-methylamino-L-alanine (BMAA), a known neurotoxin [22]. BMAA, produced by certain cyanobacteria, diatoms and dinoflagellates, poses significant risks to human health [23]. The European Food Safety Authority (EFSA) recently rejected a novel food application for an ethanolic extract of PT, citing the detection of a BMAA derivative known as 2,4-diaminobutyric acid (DAB) in one batch [24]. This underscores the importance of further research in this area. Some studies suggest that PT may indeed produce BMAA [25,26], but previous research conducted in cell cultures, mice and human studies has shown no evidence of neurotoxicity [5,6]. To advance the QPS process, it is important to quantify the BMAA content in PT biomass.

**Figure 1:**
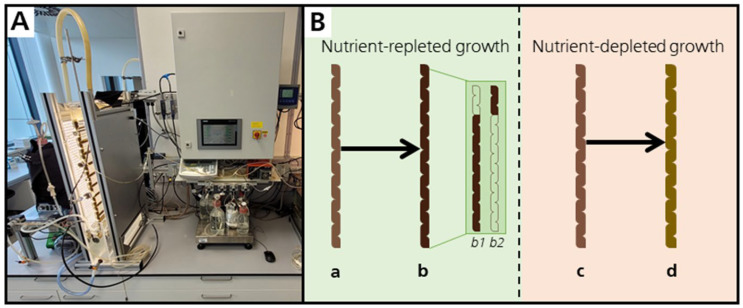
Utilized photobioreactor setup (**A**) and the applied cultivation scheme (**B**). The cultivation scheme was characterized by a nutrient-replete growth phase starting from inoculation (a). At the end of this phase (b), one fraction of the culture was harvested (b1) for the remaining culture (b2) to be used as an inoculum for the nutrient-depleted growth phase with the same initial biomass concentration as in the nutrient-replete growth phase (c). At the end of this phase, the culture was completely harvested (d).

This study presents an efficient PT biomass production process designed to deliver a nutrient-rich profile suitable for various food applications using FPA-PBRs illuminated from two sides by LED. We used a two-phase cultivation approach: an initial growth phase under nutrient-repleted conditions to optimize biomass production, followed by a second phase under nutrient-depleted conditions (Figure 1). In this way, the ingredient profile can be customized for different food applications. Throughout the process, volumetric productivity and light yield were monitored daily for overall biomass production, as well as the production of key value-added compounds, such as EPA, Fx, CRY and protein. In addition, the BMAA content was evaluated together with DAB and AEG, which are BMAA isomers [27,28], to ensure the safety and relevance of the biomass for future use as a food additive.

## 2. Results

### 2.1. Cultivation Process for Food Application

**Figure 2:**
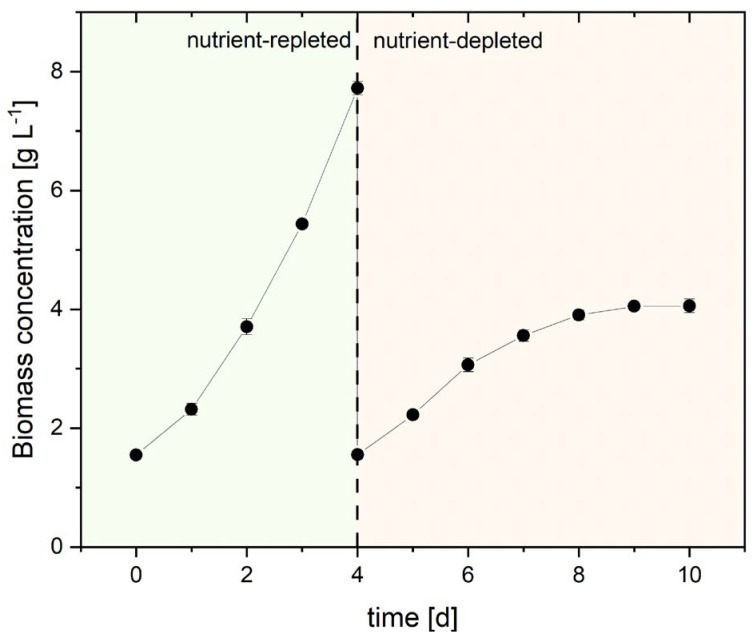
Biomass concentration during the production process. Growth phases are marked by nutrient-repleted (green) and nutrient-depleted (red) condition. Error bars display standard deviation of triplicates.

The applied cultivation procedure in this study displayed one possible opportunity to produce PT biomass with different ingredient profiles for food applications in artificially illuminated flat-plate airlift photobioreactors. The prolonged nutrient-depleted growth phase was chosen to monitor the assumed accumulation of BMMA and its derivatives during the prolonged exposure to nutrient-depletion and high light intensity. In the nutrient-repleted growth phase, the initial biomass concentration (*C_x_*) of 1.55 g_x_ L^−1^ increased to 7.72 g_x_ L^−1^ after only four days (Figure 2). In the nutrient-deplete growth phase, the initial *C_x_* of 1.55 g_x_ L^−1^ reached a plateau on day eight with only 3.90 g_x_ L^−1^. With that, the maximum volumetric biomass productivity (*Q_x_*) increased during nutrient-repleted cultivation and reached 2.29 g_x_ L^−1^ d^−1^ on day four. In turn, the maximum *Q_x_* was limited to 0.87 g_x_ L^−1^ d^−1^ on day six, resembling the second day of the nutrient-depleted growth phase. In the following days, a decrease in *Q_x_* was observed until it ceased on the last day of cultivation (Table 1). Conversely, the light conversion efficiency of the process, the biomass light yield (*LY_x_*), displayed a maximum of 1.09 g_x_ mol_photons_^−1^ on day one in the nutrient-replete growth phase, followed by a steady decline to barely calculatable values on day ten in the nutrient-depleted growth phase.

### 2.2. Ingredient Process Parameters

**Figure 3:**
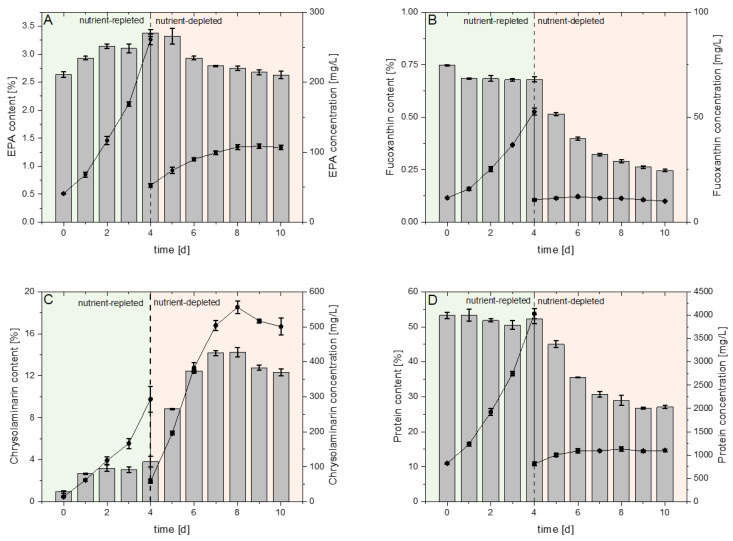
Biomass-specific content in PT biomass (bars) and respective concentrations in the culture medium (dots) of value-added ingredients eicosapentaenoic acid (EPA) (**A**), fucoxanthin (FX) (**B**), chrysolaminarin (**C**) and protein (**D**) during the production process, with respect to nutrient-repleted (green) and nutrient-depleted (red) growth condition. Error bars display standard deviation of biological triplicates from the mean.

The content of EPA in the biomass (*ω_EPA_*) increased to a maximum of 3.38% (*w*/*w*) on day four in the nutrient-replete growth phase (Figure 3A and Table 2). During the following nutrient-deplete growth phase, *ω_EPA_*, steadily decreased to a final value of 2.63% (*w*/*w*). According to *Q_x_*, the volumetric EPA productivity (*Q_EPA_*) increased during nutrient-replete growth and reached a maximum of 92.33 mg_EPA_ L^−1^ d^−1^ on day four with a subsequent decline towards EPA degradation on day ten in the nutrient-deplete growth phase. Consequentially, the EPA light yield (*LY_EPA_*) displayed a maximum of 34.19 mg_EPA_ mol_photons_^−1^ on day two, before exhibiting a descent like *LY_x_* until the end of the nutrient-depleted growth phase (Table 1). FX content (*ω_FX_*) and protein content (*ω_P_*) exhibited a maximum of 0.75% (*w*/*w*) and 53.25% (*w*/*w*) on the first day of the nutrient-replete growth phase, respectively (Figure 3B,D and Table 2). During cultivation, the chlorophyll a content (*ω_ChlA_*) was reduced from 7.30% (*w*/*w*) at t_0_ of nutrient-repleted growth over 5.97% (*w*/*w*) at the onset of nutrient-depletion to 1.93% (*w*/*w*) at the end of the cultivation (Supplementary Table S1).

The respective volumetric productivities and light yields of FX (*Q_FX_* and *LY_FX_*) and protein (*Q_P_* and *LY_P_*) matched the previously observed pattern for EPA in the nutrient-replete growth phase followed by only small alterations in the nutrient-deplete growth phase. Maximum *Q_FX_* of 15.71 mg_FX_ L^−1^ d^−1^ and *Q_P_* of 1283.28 mg_P_ L^−1^ d^−1^ were observed on day four, while maximum *LY_FX_* of 7.45 mg_FX_ mol_photons_^−1^ and *LY_P_* of 563.35 mg_P_ mol_photons_^−1^ were observed on day two. Alterations from the previously observed pattern for EPA within the nutrient-deplete growth phase were linked to an earlier onset of merely calculatable values for *Q_FX_* or *Q_P_* after seven and nine days, respectively (Table 1). The CRY content in the biomass (*ω_CRY_*) interestingly increased from the beginning to the end of the nutrient-replete growth phase from hardly detectable 0.95 to 3.80% (*w*/*w*). In the following phase, *ω_CRY_* strongly increased to a maximum of 14.24% (*w*/*w*) on day eight, followed by a decrease to 12.34% (*w*/*w*) on the last day of cultivation (Figure 3C). Interestingly, in comparison to the first days of nutrient-replete cultivation, the volumetric CRY productivity (*Q_CRY_*) on day four was two-fold increased to 134.38 mg L^−1^ d^−1^. In the nutrient-deplete phase, Q_CRY_ increased towards a maximum of 181.41 mg L^−1^ d^−1^ on day six, followed by a steady decrease until CRY accumulation was hardly detectable after nine days (Table 1). Apart from CRY, PT displayed accumulation of fatty acids during nutrient-depleted growth. The fatty acid content (*ω_FA_*) increased from 9.24% (*w*/*w*) at the end of nutrient-repleted growth to 26.03% (*w*/*w*) at the end of cultivation (Supplementary Table S1).

### 2.3. LC-MS/MS Analysis of BMAA

To assess the concentration of free BMAA and its structural isomers, AEG and DAB, in PT biomass, we utilized a targeted high-performance liquid chromatography–tandem mass spectrometry (LC-MS/MS) approach, employing amino acid derivatization with 6-aminoquinolyl-N-hydroxysuccinimidyl carbamate (AQC) and selected reaction monitoring (SRM), as described by McCarron et al. [29]. The selected reaction monitoring (SRM) transition signals of the three targeted amino acids were baseline resolved (4A). This is crucial, as the selected reaction monitoring (SRM) transitions employed for the quantification of BMAA and its structural isomers (459.1 > 171.1; 459.1 > 289.1) were identical for all three substances. The signal intensities of the SRM transitions specific to each of the three target compounds were observed at higher concentrations, but they were insufficient for quantification at trace levels, a finding that is also reported by McCarron et al. The calibration curves for BMAA, AEG and DAB in the matrix background demonstrated excellent linearity (r^2^ > 0.99) over a concentration range from 0.005 to 40 µg/mL. The limits of detection (LODs) and limits of quantification (LOQs) for the target compounds were determined using calibration standards. The LOD and LOQ were defined as the lowest concentrations, yielding signal-to-noise ratios of 3:1 and 5:1, respectively. The limit of detection (LOD) for BMAA, AEG and DAB in the absence of a matrix background (in H_2_O) was determined to be 1 pg, 1 pg and 2 pg, respectively. In the matrix background (algae extract), the corresponding LOD values for BMAA, AEG and DAB were significantly higher due to signal suppression, reaching 10 pg, 5 pg and 25 pg, respectively.

**Figure 4:**
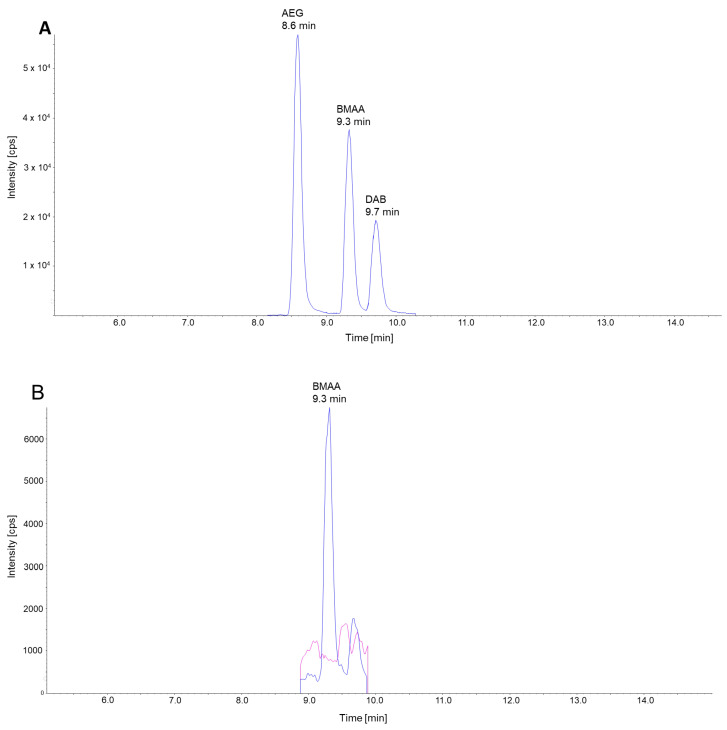
Chromatograms of LC-MS/MS analysis of BMAA and its derivatives and microalgae biomass. (**A**) Overlay of chromatograms of calibration standards of AEG, BMAA and DAB that were spiked into a microalgae sample showing the separation of AQC-derivatized AEG, BMAA and DAB signals. The retention times of AEG, BMAA and DAB signals are indicated. The chromatogram illustrates the general SRM transition *m*/*z* 459.1 > 171.1, which is applicable to BMAA, AEG and DAB. (**B**) SRM chromatogram of BMAA spiked into a microalgae sample (blue, equivalent to 50 pg on the column) and the BMAA signal in a non-spiked microalgae sample (magenta) are shown. The most intense SRM transition for BMAA *m*/*z* 459.1 > 171.1 is illustrated in the respective chromatograms.

The microalgae samples were found to be devoid of free BMAA, DAB and AEG (Figure 4B and Table 2). In the event that trace quantities of BMAA, DAB and AEG were present, the concentrations were below the specified detection limits for the method.

## 3. Discussion

This study shows the cultivation of PT and emphasizes its potential as a future component of functional foods. Due to its bioactive ingredients, such as fatty acids, carotenoids and proteins, as well as these compounds’ sustainable production compared to the State of the Art, this microalga offers promising applications in human nutrition [4]. Compared to traditional agricultural products, PT not only has a higher nutrient density, but its cultivation in photobioreactors could also help to reduce the burden on conventional resources. The absence of BMAA and its derivatives further indicates that the use of PT in food could be warranted, which is crucial for its future utilization.

By adapting and changing the cultivation conditions of PT, the content of the desired bioactive compounds can be selectively increased. Under nutrient-depleted conditions, PT biomass contains a higher content of essential nutrients, such as EPA and Fx. In contrast, the production of CRY increases significantly under nutrient-depleted conditions (Figure 3). Expectedly, the reduction in nutrient availability primarily triggered the accumulation of storage compounds such as CRY, while the overall production of EPA and Fx ceased during nutrient-depleted growth (Figure 4). This cultivation strategy, which produces two distinct biomasses, offers the possibility of utilizing PT as a food ingredient, as a dietary supplement or for the extraction of bioactive compounds.

### 3.1. Nutritional Potential of Phaeodactylum tricornutum

While the bioactive compounds in PT biomass provide nutritional value and potential health benefits, it is important to consider certain concerns. Previous studies have demonstrated the production of BMAA, with concentrations ranging from 0.20 to 1.4 μg/g dry weight after seven days of cultivation [26]. This led to the rejection of a novel food application for ethanolic PT extracts [24]. Our results show that the combination of flat-panel cultivation systems and a specific lighting configuration effectively prevents the production of BMAA in strain PT SAG 1090-1b. No BMAA or its derivatives were detected during the entire cultivation period, including the nutrient-deficient and nutrient-poor phases, as well as the extended cultivation period of up to 10 days (which is beyond the usual duration). This distinguishes our method from other approaches. We used highly sensitive detection methods that are consistent with the established literature standards [30,31]. Based on these results, the cultivation method presented here suggests that PT biomass could be safely used in food. There are currently no specific national or European-wide limits for BMAA in food, especially not for “seafood” [32]. BMAA is suspected of causing long-term damage that can lead to neurodegenerative diseases [33], so it is essential to consider BMAA as a potential food safety issue. Bioaccumulation of BMAA has been observed in seafood, particularly in species such as fish from the Baltic Sea and filter-feeding molluscs, such as mussels and oysters [34]. Although studies have not detected BMAA in commercially available spirulina products, robust quality control measures in the microalgae industry are essential to ensure food safety, especially as the consumption of microalgae-based products increases [29]. Therefore, addressing these QPS concerns could pave the way for the safe use of PT biomass in food. Previous studies in mice and humans have shown that the nutrients in PT are highly bioavailable when consumed after cell disruption [5,6]. Biomass from nutrient-repleted growth conditions, such as EPA and Fx-rich biomass, can serve as an additional nutrient source and offers potential gut health benefits [14]. From a sustainability perspective, PT offers a viable alternative as a source of EPA compared to fish oil capsules. A clinical study showed that PT as dried biomass causes comparable plasma fatty acid increases to fish oil [6]. PT could also be utilized as an additional protein source [5]. Regarding Fx from PT, it has also been shown to have antioxidant properties [35] and anti-obesity effects [8,36,37,38,39]. Previous work could show that supplementation of PT in mice fed a Western diet significantly reduced liver damage and lipopolysaccharide translocation, highlighting its potential to mitigate diet-induced metabolic stress [40]. In a clinical setup, it was further shown, with a PT extract, a possible effect on cognition and health markers in older individuals [41].

Regarding the nutrient-depleted biomass, which is CRY-rich, it also shows great potential as a dietary fiber source due to its nutrient profile. Additionally, CRY’s immunomodulatory and antioxidant properties suggest it could be used as a therapeutic agent in managing intestinal inflammation. In vivo, studies in sea bream showed that supplementation of the CRY-rich supernatant could be used as a countermeasure against intestinal inflammation due to its immunomodulatory and antioxidant effects [13]. In addition, supplementation of CRY-rich supernatant in zebrafish alleviated lipid metabolism disorders caused by a high-cholesterol diet [15]. Both PT biomasses were also tested in a clinical setup with older participants regarding low-grade inflammation and helping with healthy aging [42].

There are already several studies that have attempted to integrate PT into food. In one study, functional biscuits were produced with significantly higher amounts of microalgae, including PT, which contained more bioactive components than comparable products on the market [43]. However, the intense fishy flavor of the biscuits with PT was negatively evaluated, leading to them be rejected by consumers during tastings. In another study, PT and other microalgae, such as spirulina and *Tetraselmis*, were integrated into wheat crackers, but 60% of the participants rejected the products containing PT [44]. Despite these challenges, PT remains particularly suited for fish alternatives, as its aroma and flavor compounds resemble those of fish and macroalgae, offering potential to naturally enhance taste while providing omega-3 fatty acids [45]. To harness PT’s fishy taste for plant-based fish alternatives, it is crucial to identify suitable products and processing methods. One promising strategy is the fermentation of PT with fungi, which has been shown to reduce its intense fishy odor while enhancing sensory appeal. Fermentation transforms the undesirable fishy notes into a savory, umami-like profile, while preserving PT’s nutritional value [46]. This method holds significant potential for the development of vegetarian “fish” alternatives that are both flavorful and nutritious. Additionally, microalgae like PT could become an integral part of the future sustainable bioeconomy, offering not only the potential to fill nutrient gaps but also providing health benefits through their consumption [47].

### 3.2. Cultivation Influence on Nutrient Distribution

We underline the suitability of artificially illuminated FPA-PBRs to produce PT biomass with diverse ingredient profiles for multiple health-promoting applications. The cultivation of microalgae in general is a cost-intensive step in production, which is why efficiency is crucial for economic feasibility. For industrial applications of PT (e.g., phototrophic processes), a new production approach was investigated to reduce costs. Previous findings show that the use of artificial light is one of the main cost drivers in the production of microalgae biomass [48].

In comparison to previous studies on PT growth in FPA-PBRs illuminated from one side, we were able to reach comparable maximum biomass productivity during nutrient-repleted growth, while importantly using lower amounts of light [49]. The study [49] specified the process efficiency only in terms of photosynthetic efficiency, thereby impeding a comparison with our data. Nevertheless, the *LY_x_* during nitrogen-depleted growth was about 30% higher in our data compared to previously stated results, displaying a superior effect of two-sided illumination for biomass production [11]. This seems presumably attributed to an enhanced light supply through the two-sided illumination scheme.

While it is noteworthy to stress differences in cultivation systems and light supply, the superiority of the presented cultivation process is especially stressed concerning EPA production. The obtained data for maximum EPA content in PT biomass in our work are in good accordance with previously stated results ranging from *ω_EPA_* of 3.00 to 4.3% (*w*/*w*), regardless of the applied cultivation system or light source [48,50,51,52,53]. Nevertheless, published data for PT-based photoautotrophic volumetric EPA productivities are scarce. A previously stated study demonstrated a *Q_EPA_* of 11.8 mg_EPA_L^−1^ d^−1^ for solar-illuminated flat-panel photobioreactors [50]. Furthermore, data on the assessment of light conversion efficiency into EPA are scarce, and this scarcity might be attributed to the predominant utilization of solar light for PT cultivation and the resulting EPA production. As solar light does not account for production costs in terms of light supply, this assessment seems obsolete. The results from a previous study with an *LY_EPA_* of 17.3 mg_EPA_ mol_photons_^−1^ during solar PT cultivation put expectable values for this process parameter into perspective [50]. As stated before, artificial light is the most pronounced cost driver in PT cultivation in artificially illuminated PBR’s. Hence, *Q_EPA_* and *LY_EPA_* are of predominant importance during process development and evaluation. Compared to the previously stated values for *Q_EPA_* of 54 mg_EPA_ L^−1^ d^−1^ and *LY_EPA_* of 12.89 mg_EPA_ mol_photons_^−1^ in artificially illuminated FPA-PBR’s, our data displayed a strong increase in both values to 92.33 mg_EPA_ L^−1^ d^−1^ and 34.19 mg_EPA_ mol_photons_^−1^ [48]. This is even more pronounced due to the fact that the referenced study utilized a higher *I_spec_* of 8 µmol_photons_^−1^ g_x_^−1^ s^−1^, meaning that our results were obtained with a lower amount of light supplied. Interestingly, the FX process parameters of our work agree with the stated study.

In comparison to EPA and FX, the process parameters for CRY production diverged from the results of previous studies. In the nutrient-replete growth phase, we observed an increase in CRY content (*ω_CRY_*) from hardly detectable amounts to 3.90% (*w*/*w*), which agrees with previous findings for PT in FPA-PBRs [53]. As opposed to our work, the stated work focused solely on CRY accumulation during nutrient-depletion, making it hard to compare our results with the relevant literature. Nevertheless, and despite the common condition before the onset of nutrient-depleted growth, our results exhibited a reduced maximum *ω_CRY_* of 14.24% (*w*/*w*) compared to the 30% (*w*/*w*) previously reported [11]. The concept of the presented study only differed concerning the illumination setup and minor changes in initial biomass concentration and specific light supply, *I_spec_*. The common starting point before CRY accumulation in a nutrient-depleted growth phase, in the sense of *ω_CRY_* in the biomass, seems to be insufficient to predict the outcome of CRY accumulation during nutrient depletion. Therefore, our results point out the necessary research concerning the illumination setup and its effect on the ingredient profile. A possible explanation might be the predisposition of cultures before the nutrient-depleted growth. In our study, this was characterized by a well-monitored growth phase utilizing a high *PFD* of 466 µmol_photons_m^−2^ s^−1^ at its end. It was previously stated that a high *PFD* of up to 300 µmol_photons_m^−2^ s^−1^ led to a drastic reduction in chlorophyll content in PT [54]. As nutrient-depletion imposes a metabolic burden on cells that likely involves chlorophyll metabolism, a reduced chlorophyll content in the biomass might limit the cells’ fitness and thereby their ability to accumulate storage compounds [55].

In addition to that, the volumetric CRY productivity, *Q_CRY_*, was increased during nutrient-replete growth to 134.38 mg_CRY_ L^−1^ d^−1^, which closely resembles productivities calculated on the first day of nitrogen-depletion (145.85 mg_CRY_ L^−1^ d^−1^) and data from previous work [11]. This seems interesting, because this would allow us to produce a PT biomass with balanced amounts of health-promoting ingredients. It is important to stress that the CRY production in the nutrient-repleted growth phase would render the nutrient-depleted growth phase, in terms of light conversion efficiency inefficient, obsolete. Nevertheless, a possible explanation for the high *Q_CRY_* might be an early onset of nutrient-depletion. After all, this was prevented by the described nutrient measurement routine during nutrient-replete growth, taking into account the uncertainty of the applied methodology. Apart from nutrient starvation, high temperatures and high light intensity were proven to enhance starch accumulation in nutrient-replete conditions in other microalgae [56]. Alongside the reduced maximum (*ω_CRY_*) reached, the limitation of *Q_CRY_* might also be caused by the illumination conditions and resulting chlorophyll depletion prior to nutrient-depleted growth (Supplementary Table S1). Although this factor did not seem too pronounced in our data, the accumulation of CRY might be hindered by the strongly induced accumulation of fatty acids as energy storage (Supplementary Table S1). For the understanding of nutrient availability-dependent carbon-partitioning in microalgae, the reader is kindly referred to a comprehensive review, as this is not in the focal point here [57]. Nevertheless, the observed discrepancies in CRY accumulation in line with increased EPA productivities are far from fully understood, this leaving room for speculation and future work addressing this issue of illumination schemes in FPA-PBR cultivations. This is especially true, as differently produced PT biomasses therefore offer great therapeutic potential and could also be used to meet nutrient requirements.

The economic perspectives of a production process for nutritious PT biomass for humans must be discussed regarding the different cultivation stages because previous studies already identified a promising co-production scenario for EPA and FX in nutrient-replete growth condition [48,58]. Our new production process offers higher biomass (2.29 g L^−1^ d^−1^) and EPA productivities (92.33 mg_EPA_ L^−1^ d^−1^) while utilizing only 6 instead of 8 µmol_photons_ g_x_^−1^ s^−1^. In addition, the accumulation of CRY during the production of EPA and FX in the nutrient-replete growth phase resembles, to some extent, a PT biomass already tested in mice [13]. This further stresses the importance of our findings, as PT biomass produced in the nutrient-replete growth phase provides a good health-promoting balance of EPA, FX and CRY for human nutrition in an economically promising manner. The specialized application of CRY-rich PT biomass from nutrient-depleted growth in gut health or agriculture can still be considered [59].

## 4. Materials and Methods

### 4.1. Cultivation Procedures

In this study, *Phaeodactylum tricornutum* SAG 1090-1b, purchased from SAG culture collection of algae, Gottingen university, was used. It was cultivated in modified Mann and Myers medium containing (g L^−1^) NaCl (10.0); CaCl_2_ × 2 H_2_O (1.2); MgCl_2_ × 2 H_2_O (2.8); MgSO_4_ × 7 H_2_O (2.4); and 2% (*v*/*v*) trace element solution containing (mg L^−1^) iron citrate (253.0), Co(NO_3_) × 6 H_2_O (0.7), CuSO_4_ × 5 H_2_O (0.2), H_3_BO_0_ (600.0), MnCl_2_ × 4 H_2_O (220.0), Na_2_MoO_4_ × 2 H_2_O (25.0) and ZnSO_4_ × 7 H_2_O (33.0). Media and trace element solutions were separately sterilized by autoclaving for 20 min at 120 °C and afterwards put together. Precultures were maintained in 5 L Schott bottles with 250 µmol m^−2^ s^−1^ from not-further-specified warm-white halogen lamps. A water bath maintained a temperature of 20 °C. Preculture media contained (mg L^−1^) KNO_3_ (500.0), K_2_HPO_4_ (17.6) and KH_2_PO_4_ (22.3).

Biomass production was carried out in 6 L flat-plate airlift photobioreactors (FPA) (Subitec, Koengen, Germany) with two not-further-specified warm-white LED panels, with an automated feeding system, pH and temperature control system that was extensively described previously [48]. Cultures were inoculated from Schott bottles and allowed to adapt towards the altered illumination. During cultivation, M&M medium was utilized. Pre-sterilized nutrient solutions (g L^−1^) of NH_4_HCO_3_ (153.48), K_2_HPO_4_ (45.35) and KH_2_PO_4_ (35.80) were separately supplied by the feeding system. The supply of sufficient nutrients was routinely monitored with the semi-quantitative *QUANTOFIX^®^* system. The concentrations of the respective nutrients were always maintained above the detection limit of 10 mg NH_4_ L^−1^ and 3 mg PO_4_ L^−1^ and did not exceed 150 mg NH_4_ L^−1^ and 80 mg PO_4_ L^−1^.

Experiments were carried out with a starting *C_x_* of 1.5 g L^−1^. Cultures were illuminated with a biomass-specific light availability (*I_spec_*) of 6 µmol_photons_ g_x_^−1^ s^−1^. *I_spec_* correlates to the photon flux density (*PFD*) in µmol_photons_ m^−2^ s^−1^ to C_x_ of the culture with respect to the FPA volume of 6 L (*V_PBR_*) and its illuminated surface of 0.42 m^2^ (*A_PBR_*), as stated in Equation (1). Adaption of *PFD* was carried out once daily after measurement of *C_x_*.

(1) $$I_{spec}=\frac{PFD\;A_{PBR}}{C_{x}V_{PBR}}$$

After the nutrient-replete growth phase, cultures were diluted to 1.5 g L^−1^ for growth experiments in nutrient-depleted medium, with the same cultivation conditions as before. Nutrient depletion was verified by Hach Lange tests (LatoN, Hach Lange GmbH, Duesseldorf, Germany).

### 4.2. Dry Weight Measurement and Biomass Sampling

Culture broth was passed through a pre-dried and afterwards pre-weighted glass fiber filter by vacuum filtration. The filter was subsequently washed twice with 5 mL of tap water before being dried in a benchtop dry-scale (MA 30, Sartorius, Göttingen, Germany) and afterwards being weighted. For biomass sampling, 75 mL of culture broth was harvested by centrifugation, washed twice with distilled water and stored at −20 °C until analysis.

### 4.3. Calculations

The volumetric biomass productivity (*Q_x_*) in g L^−1^ d^−1^ was calculated from the cultivation time (*t*) in d at *t_n_* and *t*_*n*−1_ and the respective biomass concentrations (*C_x_*), according to Equation (2).

(2) $$Q_{x}=\frac{C_{x,n}-C_{x,n-1}}{t_{n}-t_{n-1}}$$

The biomass light yield, *LY_x_* (g mol_photons_^−1^), was calculated from *Q_x_* and the applied photon flux density (*PFD*) with regard to *A_PBR_* and *V_PBR_* of the cultivation system, according to (3). Moreover, *0.086* accounts for unit conversions of *PFD* from µmol_photons_ m^2^ s^1^ to mol_photons_ m^2^ d^1^.

(3) $${LY}_{x}=\frac{Q_{x}*\;V_{PBR}*\;0.086}{\;PFD*\;A_{PBR}\;}$$

Respective rates and yields of biomass ingredients were calculated from (2) and (3) by replacing *C_x_* with the respective ingredient concentration. The ingredient concentration, *C_i_* (mg L^−1^), was calculated from the respective ingredient mass percent in the biomass, *ω_i_* (% (*w*/*w*)), and the corresponding biomass concentration, *C_x_*, according to (4). The value 1000 accounts for unit conversion from g L^−1^ to mg L^−1^.

(4) $$C_{i}=\frac{\omega_{i}\;*\;C_{x\;}*1000}{100\%}$$

### 4.4. Determination of Fucoxanthin by HPLC

The Fx content, *ω_Fx_*, was determined by high-performance liquid chromatography (HPLC), utilizing a reversed-phase approach. Therefore, we deployed a Suplex pKb 100 (5 µm, 250 × 4.6 mm) column (Sigma-Aldrich Chemie GmbH, Taufkirchen, Germany) and a mobile phase consisting of solution A containing methanol/acetonitrile/2-propanol (54/44/2, *v*/*v*) and solution B containing solution A/water (85/15, *v*/*v*) for analyte separation. The applied gradient of solutions can be found elsewhere (Derwenskus et al. [48]).

### 4.5. Determination of Fatty Acid Profile and Fatty Acid Content

The determination of the fatty acids was carried out according to Frick et al. [47]. From the resulting fatty acid profile, the respective overall fatty acid content, *ω_FA_*, and the EPA content, *ω_EPA_*, in the biomass were calculated.

### 4.6. BMAA Measurement

The method was successfully developed based on Li et al. and analyzed with an LC-MS/MS [31]. The method development of BMAA is often accompanied by the detection of two other substances, 2,4-diaminobutyric acid (2,4-DAB) and aminoethylglycine (AEG)—both isomers of BMAA. The quantification of freeze-dried samples was carried out at the University of Hohenheim.

### 4.7. Amino Acid Extraction and AQC Derivatization

In total, 10 mg of microalgae samples was extracted with 1 mL of water. Cell lysis was achieved by three consecutive freeze–thaw cycles using liquid nitrogen. The samples were then incubated for 30 min at 70 °C and 1200 rpm in a thermomixer (Eppendorf, Hamburg, Germany) and sonicated for 5 min in an ultrasonic bath (Bandelin, Berlin, Germany). Subsequently, the supernatant was removed by centrifugation at 20,000× *g* for 5 min and transferred to a fresh 1.5 mL Eppendorf tube, and the samples were dried down in a vacuum centrifuge. The dried samples were reconstituted in borate buffer for AQC derivatization. For calibration, microalgae extracts that did not show MRM signals for BMAA, AEG and DAB were spiked with different levels of BMAA, AEG and DAB standards.

Amino acids were derivatized by AccQ-Tag Ultra Derivatization Kit (Waters, Eschborn, Germany) according to the manufacturer’s instructions. In brief, 6-aminoquinolyl-N-hydroxysuccinimidyl carbamate (AQC) was dissolved at 10 mg/mL in acetonitrile to prepare the AQC reagent solution. For AQC-derivatization, dried samples were reconstituted in 80 µL borate buffer. Then, 20 µL of AQC reagent solution was added, and the samples were mixed by vortexing. Subsequently, the samples were incubated for 10 min at 55 °C and 1000 rpm in a thermomixer (Eppendorf, Hamburg, Germany). Derivatized samples were stored at 4 °C until analysis by LC-MS/MS. For LC-MS/MS analysis, the samples were diluted 1:100 (*v*/*v*) with water.

### 4.8. LC-MS/MS Analysis

LC-MS/MS analysis was performed using an Agilent 1290 II UHPLC (Agilent, Waldbronn, Germany) coupled to an AB Sciex 6500+ QTRAP MS (Framingham, MA, USA) equipped with a Turbo V ion source. Chromatography separation of AQC-derivatized amino acids was achieved on an ACQUITY BEH C18 column (1.7 µm, 2.1 × 150 cm, Waters, Eschborn, Germany) maintained at 50 °C. Gradient elution was performed using water, 0.2% formic acid (solvent A) and methanol, and 0.2% formic acid (solvent B) as mobile phases at a flow rate of 0.35 mL/min. The following gradient was used: 0.0 min = 92% A; 12.0 min = 82% A; 17.0 min = 35% A; 20.0 min = 5% A; 22.0 min = 5% A; and 22.5 min = 92% A and 25.0 min = 92% A. In total, 2 µL of each standard solution and sample was injected. The 6500+ QTRAP MS was operated in positive ion mode with MRM detection to quantify BMAA, AEG and DAB. The conditions used for the 6500+ QTRAP mass spectrometer were as follows: electrospray voltage, +4500 V; source temperature, 450 °C; nebulizer gas (Gas 1), 70 psi; heater gas (Gas 2), 60 psi; curtain gas, 50 psi; and collision gas (CAD), 9 psi. Air was used as nebulizer gas and heater gas, while nitrogen was used as collision gas and curtain gas The following MRM transitions were monitored with the respective collision energies (CEs): BMAA, 459.1 > 171.1 (CE = 30) and 459.1 > 289.1 (CE = 25); AEG, 459.1 > 171.1 (CE = 30) and 459.1 > 289.1 (CE = 25); and DAB, 459.1 > 171.1 (CE = 30) and 459.1 > 289.1 (CE = 25). Transition 459.1 > 171.1 was used for quantification of BMAA, AEG and DAB, whereas transition 459.1 > 289.1 was used as a qualifier. MRM transitions specific to BMAA, AEG and DAB were observed at high concentrations with standard solutions and used for optimization of chromatography conditions to achieve the separation of BMAA, AEG and DAB. However, these MRM transitions were not observed at the LODs of BMAA, AEG and DAB. Therefore, determination of LOD and LOQ values was performed using the MRM transitions listed above.

The LOD and LOQ (Limit of Detection and Limit of Quantification) values for BMAA, AEG and DAB were determined in different matrices (without and with matrix background, such as algae extract).

BMAA in H_2_O (without matrix background):

LOD: 1 pg on the column;

LOQ: 2 pg on the column.

BMAA in algae extract (with matrix background):

LOD: 10 pg on the column;

LOQ: 20 pg on the column.

AEG in H_2_O (without matrix background):

LOD: 1 pg on the column;

LOQ: 2 pg on the column.

AEG in algae extract (with matrix background):

LOD: 5 pg on the column;

LOQ: 10 pg on the column.

DAB in H_2_O (without matrix background):

LOD: 1 pg on the column;

LOQ: 10 pg on the column.

DAB in algae extract (with matrix background):

LOD: 25 pg on the column;

LOQ: 50 pg on the column.

## 5. Conclusions

PT, if cultivated appropriately, has great potential for a wide range of food and health applications. The composition of the biomass in PT production processes can be tailored by controlling the nutrient levels during cultivation by selecting nutrient-enriched or nutrient-depleted conditions. Nutrient-repleted biomass is rich in EPA and Fx. In contrast, nutrient-depleted biomass accumulates CRY. EPA and FX exhibit antioxidative traits and can be supplied as a supplement with anti-inflammatory or anti-proliferative effects. CRY can be used as a dietary supplement to enhance positive digestion. CRY can also be used to treat intestinal inflammation. Most importantly for food and health applications, productions safety is achieved, as no toxins, such as BMAA or others, have been found in the cultivation processes. Therefore, PT biomass can be regarded as a valuable, healthy and save food ingredient in a future sustainable food production system. The application of PT biomass requires reliable production systems, ideally with artificial illumination. Under such conditions, they provide a reliable source of customized, high-quality biomass. For future work, we consider it to be highly relevant to focus on additional applications for PT biomass and to explore further possibilities for optimizing the production processes in terms of yields and costs.

## Figures and Tables

**Table 1:** Calculated process parameters volumetric productivity and light yield of PT biomass and its ingredients during the cultivation in the nutrient-repleted and nutrient-depleted growth phase. Day four displays the end of the nutrient-repleted growth phase (green) and the beginning of the nutrient-depleted growth phase (red). Values display the means of biological triplicates with standard deviation.

Time	Biomass				Eicosapentaenoic Acid				Fucoxanthin				Chrysolaminarin				Protein			
(d)	*Q_x_ ^a^*		*LY_x_ ^b^*		*Q_EPA_ ^a^*		*LY_EPA_ ^b^*		*Q_FX_ ^a^*		*LY_FX_ ^b^*		*Q_CRY_ ^a^*		*LY_CRY_ ^b^*		*Q_P_ ^a^*		*LY_P_ ^b^*	
0																				
1	0.80 ± 0.09		1.00 ± 0.11		27.36 ± 3.70		29.20 ± 3.47		4.24 ± 0.78		6.80 ± 0.72		48.55 ± 6.15		26.58 ± 3.63		408.56 ± 34.64		528.53 ± 45.79	
2	1.31 ± 0.06		1.09 ± 0.05		48.07 ± 4.39		34.19 ± 1.80		9.48 ± 1.54		7.45 ± 0.49		55.33 ± 15.32		34.66 ± 4.26		685.87 ± 39.13		563.35 ± 23.15	
3	1.74 ± 0.10		0.90 ± 0.05		52.13 ± 8.71		28.06 ± 2.34		11.46 ± 1.29		6.12 ± 0.41		48.67 ± 8.08		27.54 ± 1.68		824.87 ± 98.48		456.23 ± 30.00	
4	2.29 ± 0.07		0.81 ± 0.03		92.33 ± 9.68		27.49 ± 1.16		15.71 ± 1.82		5.54 ± 0.27		134.38 ± 19.98		30.82 ± 3.72		1283.28 ± 163.67		424.34 ± 14.13	
5	0.67 ± 0.05		0.83 ± 0.06		21.16 ± 5.24		27.62 ± 1.67		0.57 ± 0.89		4.29 ± 0.33		145.85 ± 23.41		73.55 ± 5.78		176.60 ± 6 3.61		375.68 ± 35.87	
6	0.87 ± 0.11		0.75 ± 0.09		15.93 ± 4.84		22.07 ± 2.41		0.70 ± 0.27		2.99 ± 0.31		185.83 ± 11.42		93.96 ± 11.17		87.65 ± 26.41		267.99 ± 33.41	
7	0.49 ± 0.13		0.31 ± 0.08		9.35 ± 3.23		8.64 ± 2.38		−0.69 ± 0.46		1.00 ± 0.28		121.62 ± 23.05		43.99 ± 12.42		2.28 ± 35.03		94.57 ± 23.99	
8	0.35 ± 0.07		0.19 ± 0.04		8.21 ± 2.32		5.19 ± 1.03		−0.15 ± 0.16		0.55 ± 0.10		51.92 ± 4.31		26.74 ± 4.58		37.00 ± 55.02		54.13 ± 8.32	
9	0.15 ± 0.07		0.07 ± 0.03		1.09 ± 3.43		1.99 ± 0.94		−0.69 ± 0.21		0.20 ± 0.09		−39.26 ± 18.24		9.48 ± 4.48		−44.58 ± 29.90		19.90 ± 9.37	
10	0.01 ± 0.07		0.00 ± 0.03		−1.85 ± 0.31		0.07 ± 0.91		−0.65 ± 0.18		0.01 ± 0.09		−15.74 ± 26.05		0.41 ± 4.20		14.95 ± 39.12		0.89 ± 9.46	

*^a^* *Q_i_*, volumetric biomass productivity of biomass or ingredient (g_i_ L^−1^ d^−1^). *^b^* *LY_i_*, biomass light yield of biomass or ingredient (g_i_ mol_photons_^−1^).

**Table 2:** Biomass-specific content of neurotoxic compounds β-methylamino-L-alanine (BMAA); aminoethylglycine (AEG); 2,4-diaminobutyric acid (DAB); and value-added ingredients eicosapentaenoic acid (EPA), fucoxanthin (FX), chrysolaminarin (CRY) and protein. Day four displays the end of the nutrient-repleted growth phase (green) and the beginning of the nutrient-depleted growth phase (red). Values display the mean of biological triplicates with standard deviation.

Time	BMAA	AEG	DAB	EPA	FX	CRY	Protein
(d)	*ω_BMAA_ ^a^*	*ω_AEG_ ^a^*	*ω_DAB_ ^a^*	*ω_EPA_ ^b^*	*ω_FX_ ^b^*	*ω_CRY_ ^b^*	*ω_P_ ^b^*
0	N/A	N/A	N/A	2.64 ± 0.05	0.75 ± 0.003	0.93 ± 0.13	53.25 ± 0.88
1	N/A	N/A	N/A	2.93 ± 0.03	0.68 ± 0.003	2.66 ± 0.07	53.25 ± 1.72
2	N/A	N/A	N/A	3.14 ± 0.04	0.68 ± 0.013	3.18 ± 0.28	51.85 ± 0.48
3	N/A	N/A	N/A	3.10 ± 0.08	0.68 ± 0.007	3.06 ± 0.27	50.53 ± 1.24
4	N/A	N/A	N/A	3.38 ± 0.06	0.68 ± 0.012	3.90 ± 0.41	52.21 ± 1.31
5	N/A	N/A	N/A	3.38 ± 0.06	0.68 ± 0.012	3.90 ± 0.41	52.21 ± 1.31
6	N/A	N/A	N/A	3.32 ± 0.14	0.52 ± 0.007	8.83 ± 0.06	45.04 ± 0.94
7	N/A	N/A	N/A	2.93 ± 0.03	0.40 ± 0.007	12.47 ± 0.09	35.55 ± 0.16
8	N/A	N/A	N/A	2.79 ± 0.01	0.32 ± 0.006	14.16 ± 0.24	30.71 ± 0.74
9	N/A	N/A	N/A	2.75 ± 0.04	0.29 ± 0.006	14.24 ± 0.43	28.92 ± 1.44
10	N/A	N/A	N/A	2.68 ± 0.05	0.26 ± 0.006	12.75 ± 0.25	26.76 ± 0.26

*^a^* Biomass-specific content of BMAA, AEG and DAB (pg g_x_). *^b^* Biomass-specific content of ingredients (% (*w*/*w*)). N/A, not available in accordance with the LOD of the respective compounds in H_2_O or algae matrix displayed as compound mass on the column (BMAA (1 pg/2 pg), AEG (1 pg/5 pg) and DAB (1 pg/25 pg)) (see Section 4.8).

## Data Availability

The generated data can be made available upon request to the correspondence author.

## Supplementary Materials

The following supporting information can be downloaded at https://www.mdpi.com/article/10.3390/md23020058/s1, Table S1: Biomass-specific content of total fatty acids and chlorophyll a.

## Abbreviations

AEG	Aminoethylglycine
BMAA	β-methylamino-L-alanine
ChlA	Chlorophyll a
CRY	β-1,3-glucan chrysolaminarin
DAB	2,4-diaminbutyric acid
EFSA	European Food Safety Authority
EPA	Eicosapentaenoic acid
FPA-PBR	Flat panel airlift photobioreactor
FX	Fucoxanthin
P	Protein
PT	*Phaeodactylum tricornutum*
QPS	qualified presumption of safety
*Process parameters*	
C_x_	Biomass concentration
Q	Volumetric productivity
LY	Light yield
ω_i_	Ingredient content in biomass
SRM	Selected reaction monitoring
LOD	Level of detection
LOQ	Level of quantification
