# Supplementary material for: Exploring Phaeodactylum tricornutum for Nutraceuticals: Cultivation Techniques and Neurotoxin Risk Assessment
Source: Mar Drugs. 2025 Jan 26;23(2):58. doi: 10.3390/md23020058 (PMC11857130; doi:10.3390/md23020058)
Supplement: Supplementary file 1 [file marinedrugs-23-00058-s001.zip › marinedrugs-3394336-supplementary.pdf]

marine drugs

Article

Exploring *Phaeodactylum tricornutum* for nutraceuticals: cultivation techniques and neurotoxin risk assessment

Tobias Ebbing[1,2†*], Lena Kopp[3,†], Konstantin Frick[1,2], Tabea Simon[1], Berit Würtz[4], Jens Pfannstiel[4], Ulrike Schmid-Staiger[1], Stephan C. Bischoff[3], Günter E. M. Tovar[1,2]

Table S1: Biomass-specific content of total fatty acids and chlorophyll a. Day four displays the end of the nutrient-repleted growth phase (green) and the beginning of the nutrient-depleted growth phase (red). Values display the mean of biological triplicates with standard deviation.

Time	Total Fatty Acids	Chlorophyll a
[d]	ω_{Chla} [a]	ω_{Chla} [a]
0	7.97±0.04	7.30±0.18
1	7.92±0.10	6.33±0.07
2	8.27±0.06	6.06±0.11
3	8.47±0.56	5.88±0.08
4	9.24±0.11	5.97±0.02
5	11.56±0.23	4.88±0.06
6	15.82±0.19	3.77±0.10
7	20.23±0.32	2.89±0.07
8	22.99±0.16	2.48±0.07
9	24.59±0.27	2.14±0.08
10	26.03±0.55	1.93±0.07

[a] biomass-specific content of ingredients [% $_{(w/w)}$]
